# Antifungal activity of silver/silicon dioxide nanocomposite on the response of faba bean plants (*Vicia faba* L.) infected by *Botrytis cinerea*

**DOI:** 10.1186/s40643-022-00591-7

**Published:** 2022-09-19

**Authors:** Zakaria A. Baka, Mohamed M. El-Zahed

**Affiliations:** Department of Botany and Microbiology, Faculty of Science, Damietta University, New Damietta, 34517 Egypt

**Keywords:** Antifungal activity, Silver, Silicon dioxide, Nanocomposite, *Vicia faba*, *Botrytis cinerea*

## Abstract

Silicon (Si) and its nanomaterials could help plants cope with different negative effects of abiotic and/or biotic stresses. In this study, the antifungal role of silver/silicon dioxide nanocomposite (Ag/SiO_2_NC) biosynthesized using a free-cell supernatant of *Escherichia coli* D8 was investigated for controlling the growth parameters and yield of faba bean (*Vicia faba* L.) infected by *Botrytis cinerea*. This nanocomposite was characterized using UV–Vis spectroscopy, Fourier transform-infrared (FTIR), transmission electron microscopy (TEM), zeta analysis, and X-ray diffraction pattern (XRD). Positively charged Ag/SiO_2_NC (+ 31.0 mV) with spherical-shaped silver nanoparticles (AgNPs) showed strong in vitro antifungal activity with minimal inhibition concentration (MIC) value equal to 40 ppm. In vivo experiments revealed the good resistance of Ag/SiO_2_NC-treated plants against the *B. cinerea* infection due to the increase of total phenolic content, peroxidase, and polyphenol oxidase activity. The ultrastructure of Ag/SiO_2_NC-treated plants showed normal morphology of cells including cell membranes and ellipsoidal-shaped chloroplasts with big starch grains. The concentration of silver content in Ag/SiO_2_NC-treated plants was similar to the untreated control plant indicating the low realizability of AgNPs. All of these results are promising outcomes for the application of the biosynthesized Ag/SiO_2_NC as a safe and effective antifungal agent against *B. cinerea*.

**Graphical Abstract:**

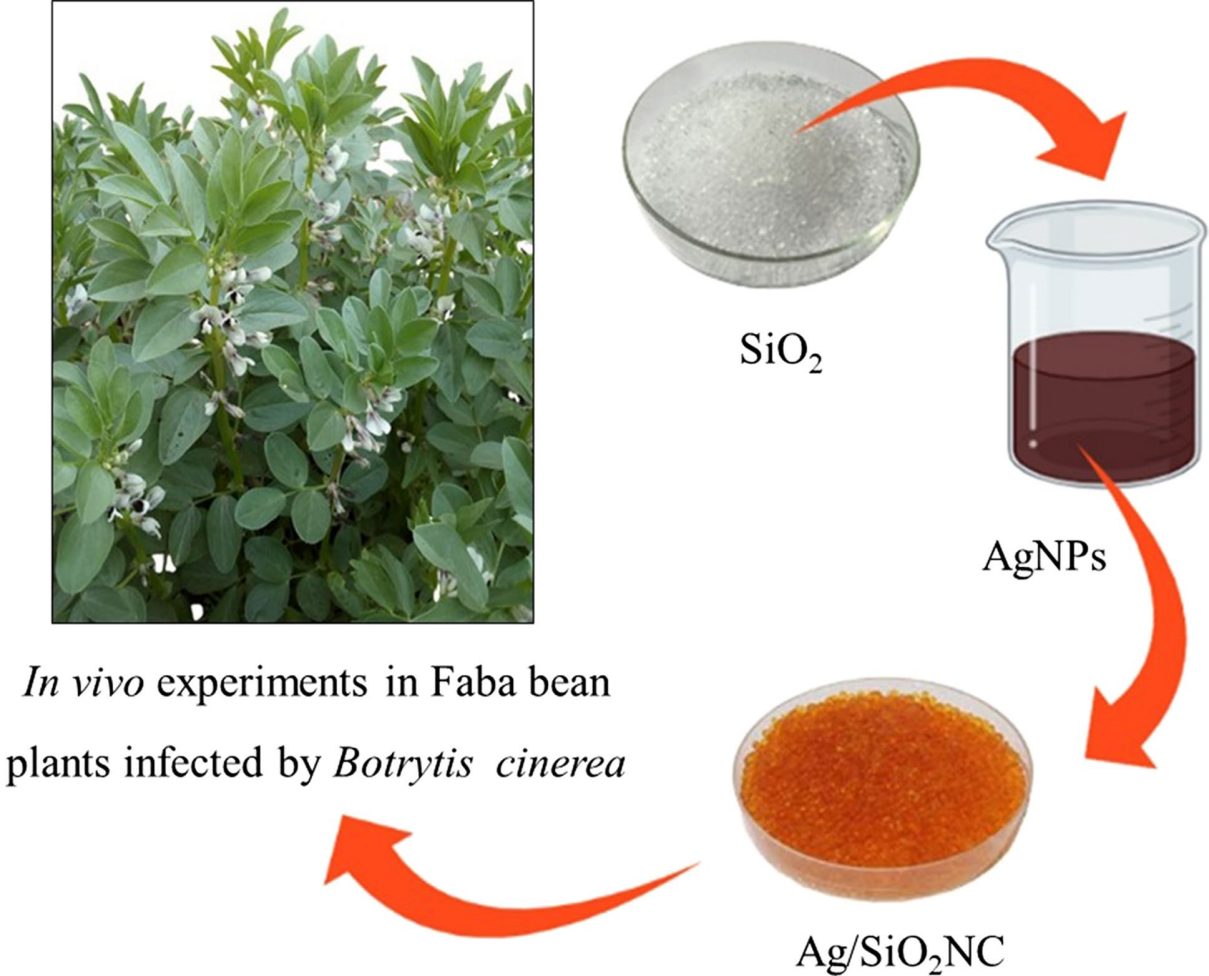

## Introduction

Nowadays, plant pathogens, especially fungi cause crop loss that threatens the food sufficiency of some countries; moreover, the huge economic losses that may be estimated at billions (Gennari et al. 2019). The faba bean plant is a multi-purpose crop that is often utilized as a common meal in poor nations and as an animal feeder in wealthy ones due to its high protein content (Brink et al. 2006). Owing to the continuous increase in demand, increasing the production of this crop is one of the agricultural goals in many nations, including Egypt, Sudan, Algeria, and others (Alaagib et al. 2022).

The faba bean is one of the most important strategic crops in Egypt, especially since it is one of the main meals on most Egyptian tables in the morning. It is considered one of the winter crops. During its growth stages, the faba bean plant needs special care to protect the crop from pests and diseases that may infect it during the growth stages and cause great losses (Sahile et al. 2008). The humid climate in Egypt (especially in winter) is suitable for the emergence and spread of many fungal diseases (Bond et al. 1994; Ouda and Zohry 2022). Chocolate spot disease caused by *Botrytis cinerea* is considered one of the most important fungal diseases that affect the faba bean crop and causes huge losses in the case of early infection (Hanounik and Hawtin 1982). This chocolate spot disease caused yield losses of 60 to 80% among susceptible cultivars and up to 34% among resistant cultivars in some African regions (Dhull et al. 2022). The disease usually appears during December and increases in January and February. Reports indicated that the *B. cinerea* chocolate spot disease is considered one of the most dangerous fungal diseases in Egypt, and threatens the productivity of faba beans, causing losses of up to 50% (Omar 2021). As it is known, the chocolate spot disease of faba bean plants consists of small distinct red–brown lesions on leaves, stems and pods (Sardiña 1929). This infection could transfer easily between the faba bean plants leading to the falling of leaves and flowers and the killing of stems. The infected pods fail to produce seeds, but if the infection occurred after the formation of the pod, the seeds formed inside will be shrunken and infected (Ellis and Waller 1974).

Thus, several studies were motivated to improve an effective elucidation for protecting food and agricultural products from this fungal infection (Hasan et al. 2020). Nanotechnology as a new technology used nanomaterials in pathogen detection, disease management and avoiding crop loss. The synthesis of nanomaterials by chemical and physical methods is highly costly and time-consuming. The biological methods for the synthesis of nanoscaled platforms include eco-friendly, uncomplicated, cost-effective, fast, and safe procedures in comparison to other chemical and physical methods (El Messaoudi et al. 2022). Nanomaterials were more proficiently and safe antifungal agents than chemical fungicides, herbicides, and fertilizers, throughout controlling their pathway and releasement rate (Li et al., 2007).

Silicon (Si) is the second most prevalent element in the earth's crust, after oxygen, and has a critical role in the growth, metabolism, defence and development of various crops (Mukarram et al. 2021). Silicon was reported as a strong inhibitor for fungal spore germination, germ tube elongation, and mycelial growth (Liu et al. 2010). On a similar trend, Si nanomaterials have been reported to have stronger impacts on plant growth and physiology than bulk Si (Tripathi et al. 2016). Besides Si, silver nanoparticles (AgNPs) are now one of the most commercialized nanomaterials possessing applications in several agricultural products as they kill plant pathogenic fungi and bacteria (Singh et al. 2015). The combination of Si and AgNPs will offer up new possibilities for using Si nanomaterials as a growth elicitor in a variety of commercial crops.

Therefore, the objectives of this study were to biosynthesize and investigate the antifungal activity of the silver/silicon dioxide nanocomposite (Ag/SiO_2_NC) against *B. cinerea*, to our knowledge, as a first in vivo study of such nanocomposite, and to evaluate the control efficacy of chocolate spot disease of *Vicia faba* L. caused by *B. cinerea*.

## Materials and methods

### Materials and reagents

Silicon dioxide (SiO_2_) (granular, ≥ 99.9%), silver nitrate (AgNO_3_, crystals, ≥ 99.9) and culture media were purchased from Sigma–Aldrich Chemie, Steinheim, Germany. The Agricultural Research Center in Giza, Egypt, provided seeds of the faba bean cultivar (Giza 429) and the fungicide, Dithane M-45 (ethylene bis dithiocarbamate at 80%, 16 percent manganese, and just 2% zinc). *Escherichia coli* D8 (AC: MF062579) and *B. cinerea* (AC: KP151604) were provided by the Laboratory of Microbiology, Faculty of Science, Damietta University, Egypt. *Botrytis cinerea* was cultivated on faba bean dextrose agar (FDA) and incubated for 7 days at 25 °C.

### Instruments

The absorbance measurements were performed using a UV–Vis spectrophotometer (Beckman DU-40, USA). X-ray X' Pert powder diffractometer (Philips, D8-Brucker Model), fitted with Ni filter and Cu k-radiation (= 1.5418) at 40 kV and 30 mA, was used to record the Ag/SiO_2_NC X-ray diffraction (XRD) pattern. Fourier transform-infrared (FTIR) spectra were carried out using a KBr disc (KBr pellet) on a JASCO FTIR-410 spectrometer in the 4000–400 cm-1 region. Transmission electron microscopy (TEM, JEOL JEM-2100, Japan) and zeta potential analyses and were carried out at the Electron Microscope Unit, Mansoura University, Egypt. All previous instruments were used to characterize the biosynthesized nanocomposite. An atomic spectrometer (PerkinElmer, PinAAcle-500, UK) was used in measuring the concentration of silver content in Ag/SiO_2_NC-treated plants.

### Biosynthesis of Ag/SiO_2_NC

Ag/SiO_2_NC was prepared according to Sadeghi et al. (2013) and modified by El-Zahed et al. (2022). Briefly, AgNPs were biosynthesized in the presence of sunlight by mixing free-cell supernatant of *E. coli* D8 (filtered from overnight bacterial growth, inoculated by a 0.5 McFarland standard, 1–2 × 10^8^ CFU/ml) with 1.5 mM of AgNO_3_ solution (1:1 v/v). After the formation of the brown colour (the first indicator for AgNPs formation), the reaction was added to another beaker that included 100 g of SiO_2_. The whole solution was stirred for 30 min until the SiO_2_ granules were brown in colour. Finally, the Ag/SiO_2_NC granules were centrifuged, washed 3 times with distilled water and collected. The product was dried at 50 °C for 24 h and 185 °C for an additional 5 h. Dried Ag/SiO_2_NC was characterized using UV–Vis spectroscopy, FTIR, TEM, zeta potential analysis, and XRD.

### Minimal inhibition concentration

Minimal inhibition concentration (MIC) of Ag/SiO_2_NC against *B. cinerea* was used to investigate the antifungal activity of Ag/SiO_2_NC in comparison to its bulk materials. Faba bean dextrose broth (FDB) medium was prepared, distributed into different flasks, and autoclaved. Different concentrations of AgNO_3_, SiO_2_ and Ag/SiO_2_NC (1–200 ppm) were added into FDB flasks separately and aseptically. Each flask was inoculated by a 5 mm *B. cinerea* disc (7 days growth) and incubated at 25 °C for 7 days. The biomass was collected after the incubation period by filtration using dry known weighted Whatman filter paper No. 1 (dried at 80 °C for 2 h), followed by vigorous washing with sterile distilled water to eliminate any medium components. The dry weight of fungal biomass was calculated, and the % inhibition of fungal growth was determined relative to the control. Dithane M-45 was made similarly and used as a positive control. The inhibition percentage (I%) was calculated according to the Tops and Wain equation (1957) as follows:

$${\text{I}}\% \, = \, \left( {{\text{A}} - {\text{B}}} \right)/{\text{A }} \times { 1}00,$$

where I%, inhibition percentage; A, dry weight of fungal biomass in the control; and B, dry weight of fungal biomass in treatment.

### Field experiments

The experiment aimed to compare Ag/SiO_2_NC antifungal activity to that of a widely used fungicide (Dithane M-45). As antifungal agents, Ag/SiO_2_NC and Dithane M-45 were used at MIC doses. Faba bean seeds were surface sterilized in 0.01% mercuric chloride for 3 min, then washed several times with sterilized water to eliminate surplus disinfectant. Clay and sand were mixed in a 2:1 v/v ratio (Million et al. 1987) and autoclaved for 30 min. Three sterilized seeds per pot were planted in 20-cm plastic pots with autoclaved potting mix. Plants were grown at an average temperature of 22 °C in the light and 10 °C in the dark from half December to the beginning of April. Seedlings were irrigated and kept in the field growth conditions. Seedlings were watered with antifungal agent solutions after 28 days from planting, and after 7 days, the treatment was repeated. Plants were infected with 2.5 × 10^5^ spore/ml of *B. cinerea* spore suspension when spraying the plants until wetness, then covered with transparent polyethylene bags. For comparative purposes, a control group was left without infection and treatment. To estimate growth parameters, leaf samples were obtained 72 days after planting. After 130 days, the final harvest was carried out to acquire the yield and estimate growth characteristics.

### Analyses of growth and yield parameters

Shoot and root lengths, fresh and dry weight of shoots, shoot diameter, leaf area, root fresh and dry weight, number of nodes, number of legumes, legume air dry and oven-dry weight, number of seeds per legume, fresh and dry mass of seeds, the weight of 100 seeds were all measured for each harvest after 70 days from planting. Dry weights were recorded after drying the samples at 80 ºC for 48 h in a hot air oven until constant weight. All the weights were measured in grammes (g). Harvest index, mobilization index, crop index and relative seed yield were also calculated according to Hall et al. (2013) as follows:

$${\text{Shoot or root distribution }} = {\text{ Fresh mass }}/{\text{ length,}}$$

$${\text{Shoot or root density }} = {\text{ Dry mass/length,}}$$

$$\mathrm{Harvest\, index }= \frac{\mathrm{Seed}\, \mathrm{weight}(g)/\mathrm{plant}}{\mathrm{Straw}\, \mathrm{weight}(g)/\mathrm{plant}} \times 100,$$

$$\mathrm{Mobilization\, index }= \frac{\mathrm{Crop}\, \mathrm{weight} (g)/\mathrm{plant}}{\mathrm{Straw}\, \mathrm{weight} (g)/\mathrm{plant}} \times 100,$$

$$\mathrm{Crop \,index }= \frac{\mathrm{Seed} \mathrm{weight} (g)/\mathrm{plant}}{\mathrm{Seed}\, \mathrm{weight} (g)/\mathrm{plant} + \mathrm{Straw} \, \mathrm{weight} (g)/\mathrm{plant}} \times 100,$$

$$\mathrm{Relative\, seed \,yield }= \frac{\mathrm{Yield}\, \mathrm{in}\, \mathrm{treatment}}{\mathrm{Yield}\, \mathrm{in}\, \mathrm{control}} \times 100.$$

### Estimation of proline

The method described by Snell and Snell (1959) was used to calculate proline. A combination of 4 ml of syrupy phosphoric acid 1:1 dilution and 6 ml of glacial acetic acid was employed as a reagent, along with 0.25 g of ninhydrin, which was heated to 70 ºC to full solubility. A blank utilizing the acid combination without ninhydrin was generated by pipetting one ml of concentrated water extract into a Quickfit tube, adding 1.0 ml of glacial acetic acid, then 1.0 ml of the reagent at the same time. There was also a reagent blank prepared. The samples and blanks were heated at 100 ºC for 60 min. The tubes were then filled with 1.0 mL glacial acetic acid and allowed to cool to room temperature. With glacial acetic acid, the volume in each tube was adjusted to 5 ml. The optical density of the generated colour was determined spectrophotometrically within one hour at 515 nm.

### Estimation of total phenols

According to Singleton and Rossi (1965), total phenols in plants were determined as follows: T = [c × v/m] × 100, where T represents total phenolic content (mg catechol/100 g fresh weight), c represents catechol concentration, v represents volume utilized (ml), and m represents plant mass (g). The total phenolic content of plants infected with *B. cinerea* was determined using a spectrophotometric technique at 650 nm.

### Estimation of peroxidase activity (POD)

At 4 °C, 0.5 g of leaf material was homogenized in a mortar with 30–40 ml of 0.02 M phosphate buffer (pH7), filtered and centrifuged at 4000 rpm for 10 min. The extract was then made up to 100 ml with the buffer. 0.1 ml from the extract was added to the reaction mixture of 0.5 ml 1% H_2_O_2_, 3 ml pyrogallol phosphate buffer (0.05 M pyrogallol in 0.1 M phosphate buffer, pH6). The production of purpurogallin caused a rise in absorbance at 420 nm, which was used to measure POD activity (Devi 2000). One enzyme unit is one per g of fresh material per min.

### Estimation of polyphenol oxidase activity (PPO)

Using the previously prepared extract for POD estimation, the production of purpurogallin was used to measure PPO (Devi 2000). About 1 ml of the extract was added to 2 ml of 0.02 M phosphate buffer (pH 7) and 1 ml 0.1 M pyrogallol to the reaction mixture. Then, 1 ml of 2.5 N H_2_SO_4_ was added to the reaction mixture after 1 min of incubation at 25 ºC. A unit of enzyme is one per g of fresh material per min.

### Estimation of total protein of faba bean seeds

The protein content of faba bean seeds was estimated according to Bradford (1976). A known weight from fresh seeds was macerated in a mortar with 2 ml of extraction buffer (0.2 M Tris–HCl, pH 6.8, 2% SDS, and 10% sucrose) and centrifuged at 4000 rpm for 15 min. The absorbance was measured at 595 nm against a blank made from 0.1 ml of the appropriate buffer plus a 5-ml protein reagent. Using bovine serum albumin solution as the standard protein, the amount of protein in the samples was determined from the standard curve.

### Estimation of silver concentration content

The faba bean leaves and seeds were digested for 4 h with a solution of 10 ml concentrated nitric acid, 4 ml perchloric acid (60%), and 1 mL concentrated sulfuric acid. The digested contents were diluted with distilled water and filtered through a Whatman no. 42 filter before being measured for silver total mass using the atomic spectrometer (Issac and Johnson 1975).

### Ultrastructural study

According to Hayat (1989), the processing of specimens for transmission electron microscope (TEM) was carried out by cutting faba bean leaves into small pieces and fixed with 2.5% glutaraldehyde in 0.1 M phosphate buffer for 24 h at room temperature. The specimens were washed with phosphate buffer and then fixed with 1% osmium tetroxide for 90 min at 4 °C and the dehydration was done by ethanol gradient and then replaced by 100% acetone. The specimens were impeded in resin (Epon 812, Switzerland) followed by sectioning into semi-thin and ultrathin sections with the help of an Ultramicrotome (RMC PT-XL Power Tome Ultra microtome). The ultrathin sections were stained by uranyl acetate followed by lead citrate and examined under a TEM at 160 kV (JEOL-JEM 2100) at 80 kV at the Electron Microscopy Unit, Mansoura University, Egypt.

### Statistical analysis

The data were analysed using SPSS version 18 and ANOVA. The *p* < 0.05 significance threshold was used. All the experiments were carried out three times. The mean and standard error (SE) were used to express all the data.

## Results

### Characterization of biosynthesized Ag/SiO_2_NC

**Fig. 1:**
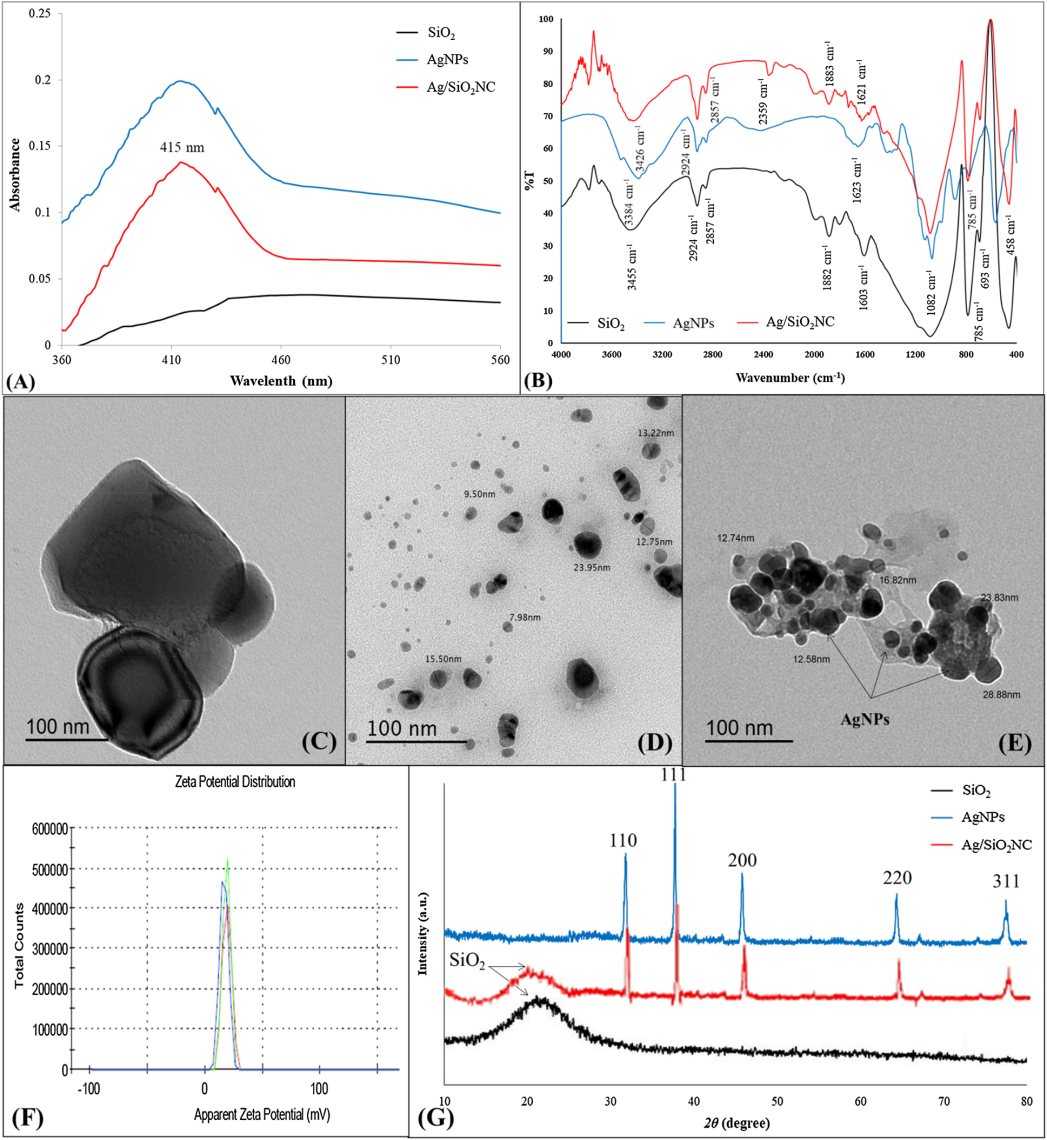
Characterization of Ag/SiO_2_NC. **A** The UV–vis spectra of SiO_2_, AgNPs and Ag/SiO_2_NC. **B** The FTIR spectra of SiO_2_, AgNPs and Ag/SiO_2_NC. **C** TEM of SiO_2_, **D** AgNPs and **E** TEM of Ag/SiO_2_NC with bars scale = 100 nm. **F** Zeta potential measurement of Ag/SiO_2_NC. **G** The XRD patterns of SiO_2_, AgNPs and Ag/SiO_2_NC

The colour of SiO_2_ granules changed from white to brown, suggesting that Ag/SiO_2_NC had been formed. Figure 1A shows the UV–Vis spectra of SiO_2_, AgNPs and Ag/SiO_2_NC. There are no apparent absorption peaks at 400–750 nm for pure SiO_2_; however, a clear absorption peak develops at about 415 nm for AgNPs and Ag/SiO_2_NC particles, which is the typical absorption of nanosilver. FTIR spectra (Fig. 1B) of Ag/SiO_2_NC annealed between 400 and 4000 cm^−1^. Water bands corresponding to bending vibrations were identified in all the IR spectra, indicating that the powdered materials are hygroscopic. Si–O–Si and Si–OH absorptions are responsible for the strong bands detected at 1082, 785, and 458 cm^−1^. At roughly 462 and 693 cm^−1^, there is also a little amount of absorption owing to the Si–O–Ag connections stretching. The presence of the band in Ag/SiO_2_NC suggests that AgNPs and oxygen bound to silica are bonded. The morphology of AgNPs was investigated using the TEM (Fig. 1C, D, and E). AgNPs are found both on the surface of the silica and within the matrix. The TEM micrograph revealed a wide size dispersion of spherical AgNPs with diameters ranging from 12 to 29 nm. On the one hand, the coupling agent allows AgNPs with tiny particle sizes and large surfaces to mix well with the polymer matrix in Ag/SiO_2_NC. Zeta potential analysis (Fig. 1F) confirmed the positive charge of the biosynthesized Ag/SiO_2_NC (+ 31.0 mV). SiO_2_ and Ag/SiO_2_NC XRD patterns (Fig. 1G) were investigated. There were no additional diffraction peaks for pure silica particles. The amorphous silica characteristic diffraction peaks appeared at 15–25° (Wu et al. 2016). This amorphous character of SiO_2_ confirm its high adsorbing function to AgNPs. The XRD patterns of AgNPs and Ag/SiO_2_NC showed peaks at 5 angles of 31.88°, 37.92°, 44.1°, 64.20° and 78.2° which corresponded to the reflections of the (110), (111), (200), (220) and (311) crystalline planes of AgNPs’ face-centred cubic (FCC) structure, suggesting that the coatings of AgNPs have crystallized well on the surfaces of amorphous SiO_2_ which might decrease the amorphous peak of Ag/SiO_2_NC and made it little broader and less than bulk SiO_2_ (Nguyen and Nguyen 2020; Kadhim et al. 2022). This coincides well with the XRD pattern of solo AgNPs particles. In addition, the XRD does not show the typical silver oxide peaks. It means that the Ag/SiO_2_NC coverage is pure AgNPs, not silver oxide or other contaminants.

The size of nanoparticles was estimated from the Debye Scherrer’s formula: *d* = 0.89λ/(*β* cos *θ*), where λ is the X-ray wavelength, *β* is the full-width at half-maximum of the X-ray diffraction peak and *θ* is the diffraction angle (Birks and Friedman 1946). The estimated mean diameter of nanoparticles size was 20.4 nm in good agreement with those observed in TEM results.

### Minimal inhibition concentration of Ag/SiO_2_NC against *B. cinerea*

**Fig. 2:**
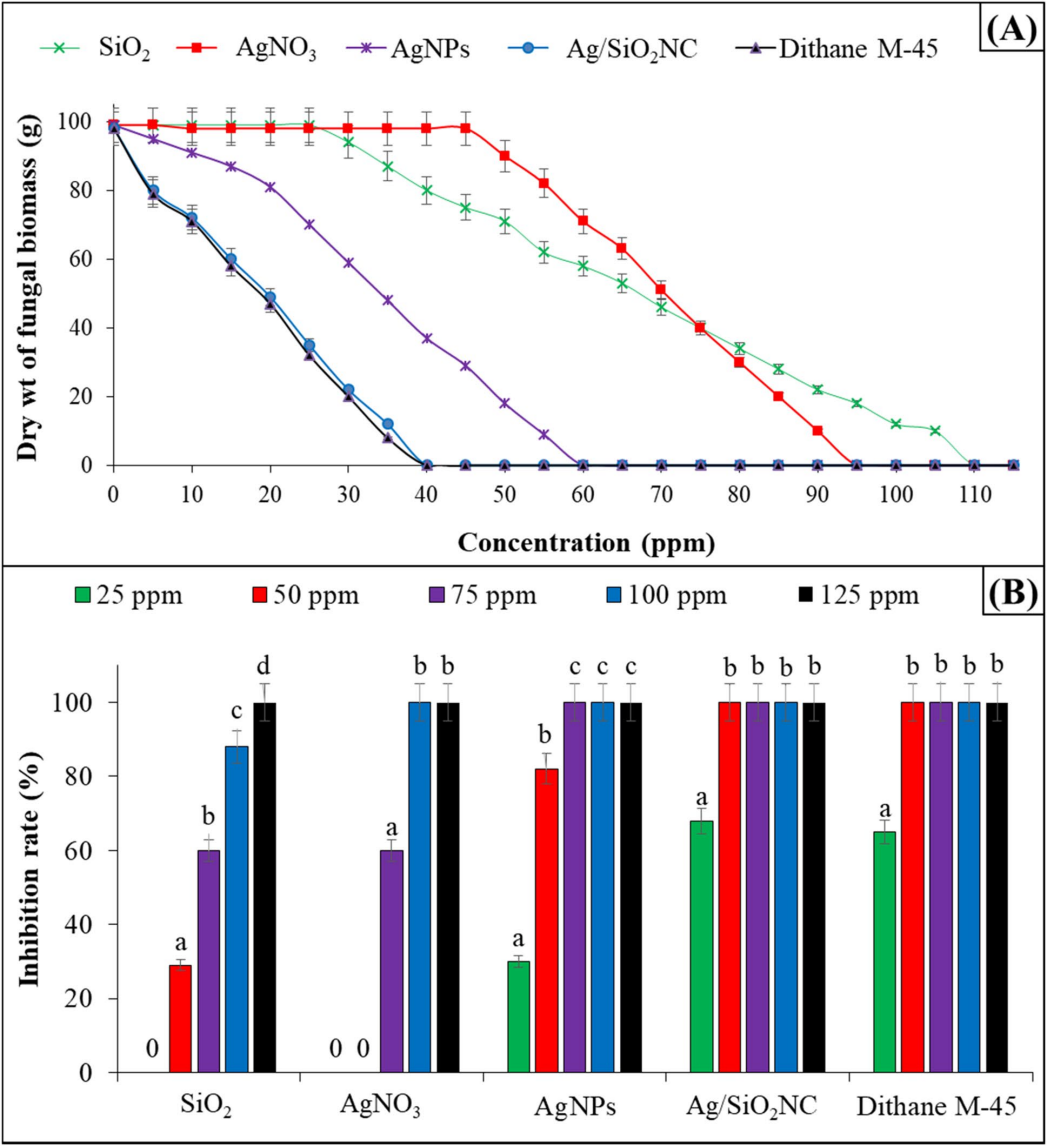
**A** The minimal inhibition concentration and **B** the inhibition percentage of AgNO_3_, SiO_2_, AgNPs, Ag/SiO_2_NC and Dithane M-45 against *B. cinerea*

Figure 2 shows that 40 and 60 ppm of Ag/SiO_2_NC and AgNPs, respectively (MIC value) and other high concentrations have a better fungicidal effect than lower ones. In a dose-dependent manner, Ag/SiO_2_NC demonstrated a good antifungal action against *B. cinerea*. While the MIC values of AgNO_3_ and SiO_2_ were 95 and 110 ppm, respectively. Although the Egyptian agricultural ministry recommended Dithane M-45 as a very strong antifungal agent against the development of chocolate spot disease caused by *B. cinerea*, the inhibitory rate values of Ag/SiO_2_NC were close to Dithane M-45 MIC values, indicating the nanocomposite's high efficiency against *B. cinerea*. Also, Ag/SiO_2_NC revealed more antifungal potential against *B. cinerea* than AgNO_3_ and SiO_2_.

### Field growth condition experiments

**Fig. 3:**
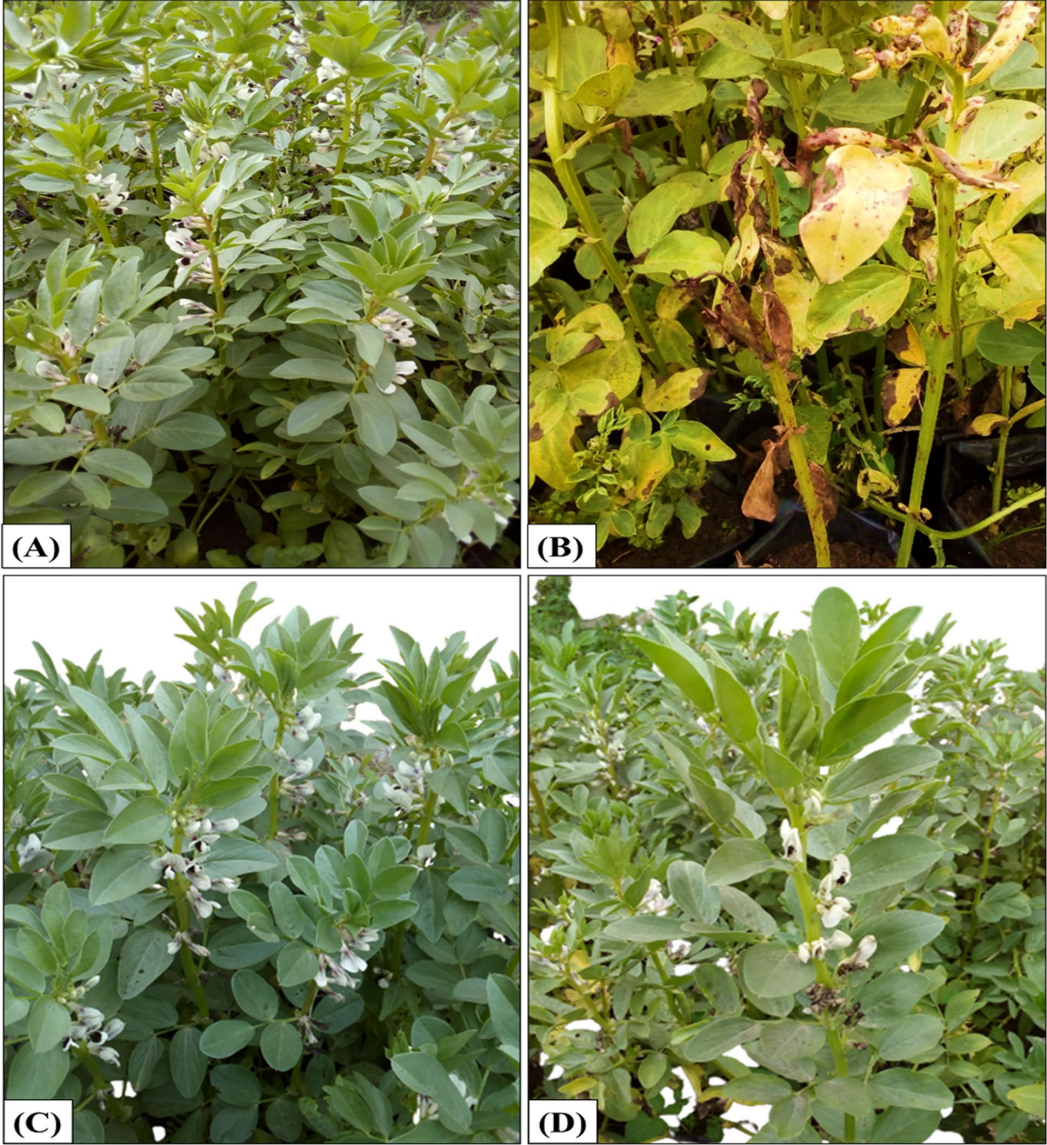
Field growth condition experiments. **A** Non-infected and untreated control. **B** Infected and untreated control. **C** Infected and treated by Ag/SiO_2_NC at 40 ppm. **D** Infected and treated by Dithane M-45 at 40 ppm

In comparison with the Ag/SiO_2_NC-treated, Dithane M-45-treated and control plants, chocolate spot symptoms emerged in the untreated and infected plants with *B. cinerea* (Fig. 3). The affected plants had necrotic flecks on their leaves, which were the typical symptoms of the chocolate spot disease. Furthermore, the untreated infected faba bean plants' green shoot length and flowering rate were lower than other plants.

### Analyses of growth and yield parameters

Table 1 reveals that pathogen reduced root biomasses (fresh and dry masses), root length, root density, root dispersion, and root/shoot ratio when compared to control values. In the presence or absence of pathogen, the fungicide induced a significant reduction in all plant growth parameters except root distribution. Root biomasses, length, and root/shoot ratio all increased significantly after using Ag/SiO_2_NC.

**Table 1:** Effect of Ag/SiO_2_NC and Dithane M-45 on the growth vigour of faba bean plants root

Treatments^a^	Fresh mass (g)	Dry mass (g)	Length (cm)	Distribution (g/cm)	Density (g/cm)	Root/shoot ratio
Non-infected and untreated control	8.03 ± 0.57c^b^	1.91 ± 0.14c	16.52 ± 0.96c	0.49 ± 0.06c	0.12 ± 0.04b	0.24 ± 0.06b
Infected and treated by Ag/SiO_2_NC	4.20 ± 0.19a	1.43 ± 0.31b	10.89 ± 0.48a	0.39 ± 0.03a	0.13 ± 0.01b	0.19 ± 0.02a
Infected and treated by Dithane M-45	10.23 ± 0.72d	2.30 ± 0.19d	20.60 ± 0.18d	0.50 ± 0.05c	0.11 ± 0.01b	0.27 ± 0.03b
Infected and untreated control	6.50 ± 0.33b	0.88 ± 0.12a	14.30 ± 0.13b	0.45 ± 0.12b	0.06 ± 0.04a	0.22 ± 0.09a

^a^The treatments including control, infected plants, Ag/SiO_2_NC, Dithane M-45

^b^Values within each column, means ± SE (*n* = 9) followed by a similar letter are not significantly different at *p* ≤ 0.05 using Tukey–Kramer HSD test

Table 2 shows the differences in shoot growth vigour of the variously treated faba bean plants. The results revealed that untreated infected plants and fungicide-treated plants had considerable reductions in the shoot growth vigour of faba bean. Furthermore, as compared to control values, fungicide produced substantial reductions in shoot biomasses (fresh and dry weights), length, diameter, density, and leaf area. The treatment of faba bean plants with Ag/SiO_2_NC, on the other hand, resulted in a considerable rise in these parameters.

**Table 2:** Effect of Ag/SiO_2_NC and Dithane M-45 on the growth vigour of faba bean plants shoot

Treatments^a^	Fresh mass (g)	Dry mass (g)	Length (cm)	Diameter (mm)	Distribution (g/cm)	Density (g/cm)	Leaf area (cm^2^)	No. of nodes
Non-infected and untreated control	48.60 ± 0.53c^b^	7.35 ± 1.31c	69.54 ± 1.02 c	7.11 ± 0.11c	0.70 ± 0.08b	0.11 ± 0.05b	41.20 ± 0.57c	20 ± 0.19b
Infected and treated by Ag/SiO_2_NC	30.33 ± 0.37a	4.00 ± 1.58a	56.00 ± 1.00a	5.51 ± 0.02a	0.54 ± 0.09 a	0.07 ± 0.03a	28.60 ± 0.36a	16 ± 0.14a
Infected and treated by Dithane M-45	59.80 ± 1.29d	9.50 ± 0.47d	76.20 ± 0.88d	7.69 ± 0.34 d	0.79 ± 0.06c	0.13 ± 0.06c	44.50 ± 0.42d	23 ± 0.22c
Infected and untreated control	46.50 ± 0.68b	6.90 ± 0.25b	67.50 ± 1.14b	6.52 ± 0.01 b	0.69 ± 0.04b	0.10 ± 0.01b	39.60 ± 1.20b	19 ± 0.09b

^a^The treatments including control, infected plants, Ag/SiO_2_NC, Dithane M-45

^b^Values within each column, means ± SE (*n* = 9) followed by a similar letter are not significantly different at *p* ≤ 0.05 using Tukey–Kramer HSD test

The impact of Ag/SiO_2_NC and Dithane M-45 on yield components of faba bean infected with *B. cinerea* is shown in Tables 3, 4. In comparison to control plants, infected plants and Dithane M-45-treated plants had a significant decrease in all yield components of faba bean plants, including a noticeable reduction in straw production per plant, relative seed yield, and biological yield. On the other hand, Ag/SiO_2_NC caused an enormous increase in practically all yield components of faba bean.

**Table 3:** Effect of Ag/SiO_2_NC and Dithane M-45 on yield and yield components of faba bean plants

Treatments^a^	No. of pods/plant	No. of seeds/pod	Seed biomass (g)		Pod biomass (g)		100 seeds wt (g)	Seed yield/plant (g)
			Fresh	Dry	Fresh	Dry		
Non-infected and untreated control	9.50 ± 0.92c^b^	3.60 ± 0.29c	0.93 ± 0.03a	0.81 ± 0.01a	4.10 ± 1.43c	3.60 ± 0.14b	96.45 ± 2.11b	34.20 ± 0.13c
Infected and treated by Ag/SiO_2_NC	6.30 ± 1.25a	2.50 ± 0.08a	1.40 ± 0.03b	1.20 ± 0.05b	2.60 ± 1.05a	2.10 ± 0.03a	76.20 ± 1.45a	15.75 ± 0.59a
Infected and treated by Dithane M-45	10.50 ± 1.31d	5.30 ± 0.36d	1.80 ± 0.06c	1.70 ± 0.05c	5.10 ± 0.99d	4.40 ± 0.06c	101.00 ± 1.13c	55.65 ± 0.04d
Infected and untreated control	8.10 ± 0.22b	3.40 ± 0.17b	0.87 ± 0.03a	0.80 ± 0.07a	3.20 ± 0.67b	3.60 ± 0.16b	95.10 ± 2.03b	27.54 ± 1.44b

^a^The treatments including control, infected plants, Ag/SiO_2_NC, Dithane M-45

^b^Values within each column, means ± SE (*n* = 9) followed by a similar letter are not significantly different at *p* ≤ 0.05 using Tukey–Kramer HSD test

**Table 4:** Effect of Ag/SiO_2_NC and Dithane M-45 on the yield and yield components of faba bean plants

Treatments^a^	Straw yield/plant (g)	Harvest index	Mobilization index	Crop index
Non-infected and untreated control	22.70 ± 1.03c^b^	1.51 ± 0.03a	2.10 ± 0.11b	0.60 ± 0.03 a
Infected and treated by Ag/SiO_2_NC	11.30 ± 1.21a	1.40 ± 0.06a	1.70 ± 0.04a	0.58 ± 0.03a
Infected and treated by Dithane M-45	30.20 ± 0.23d	1.84 ± 0.14c	2.40 ± 0.15c	0.65 ± 0.03c
Infected and untreated control	16.40 ± 0.14b	1.68 ± 0.06b	2.10 ± 0.11b	0.63 ± 0.14b

^a^The treatments including control, infected plants, Ag/SiO_2_NC, Dithane M-45

^b^Values within each column, means ± SE (*n* = 9) followed by a similar letter are not significantly different at *p* ≤ 0.05 using Tukey–Kramer HSD test

### Effect of Ag/SiO_2_NC on proline content

**Fig. 4:**
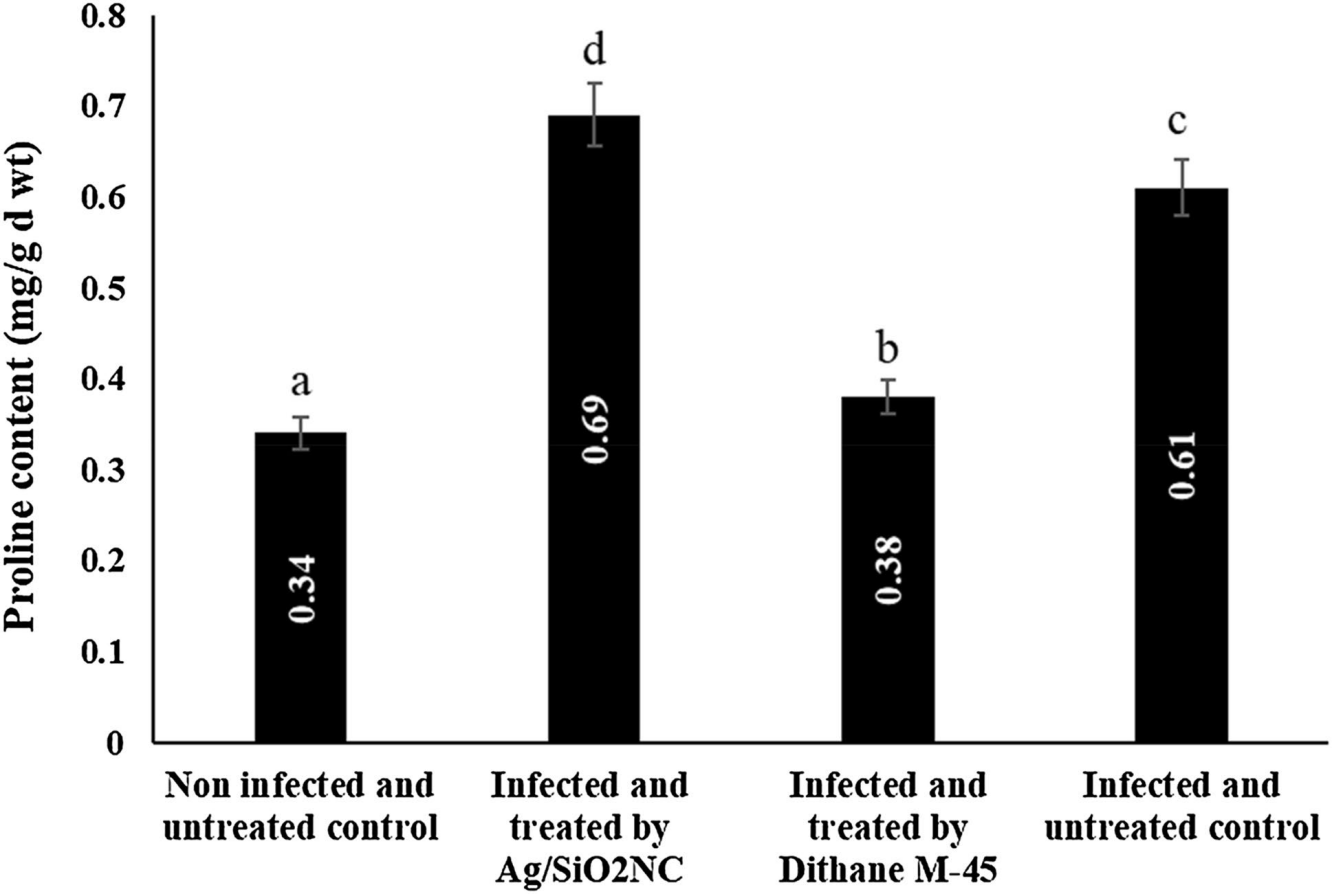
The proline content in leaf extract of faba bean plants (non-infected/infected) in the presence or absence of Ag/SiO_2_NC or Dithane M-45. Vertical bars represent the SE. Means denoted by similar letter are not significantly different at *p* ≤ 0.05 using Tukey–Kramer HSD test

In comparison to the non-infected untreated control plants, infection of faba bean plants with *B. cinerea* increased proline concentration (Fig. 4). Furthermore, treatment with Ag/SiO_2_NC (MIC, 40 ppm) resulted in a considerable rise in proline concentration. As a result, Ag/SiO_2_NC might help faba bean plants become more physiologically resistant.

### Effect of Ag/SiO_2_NC on total phenols

**Fig. 5:**
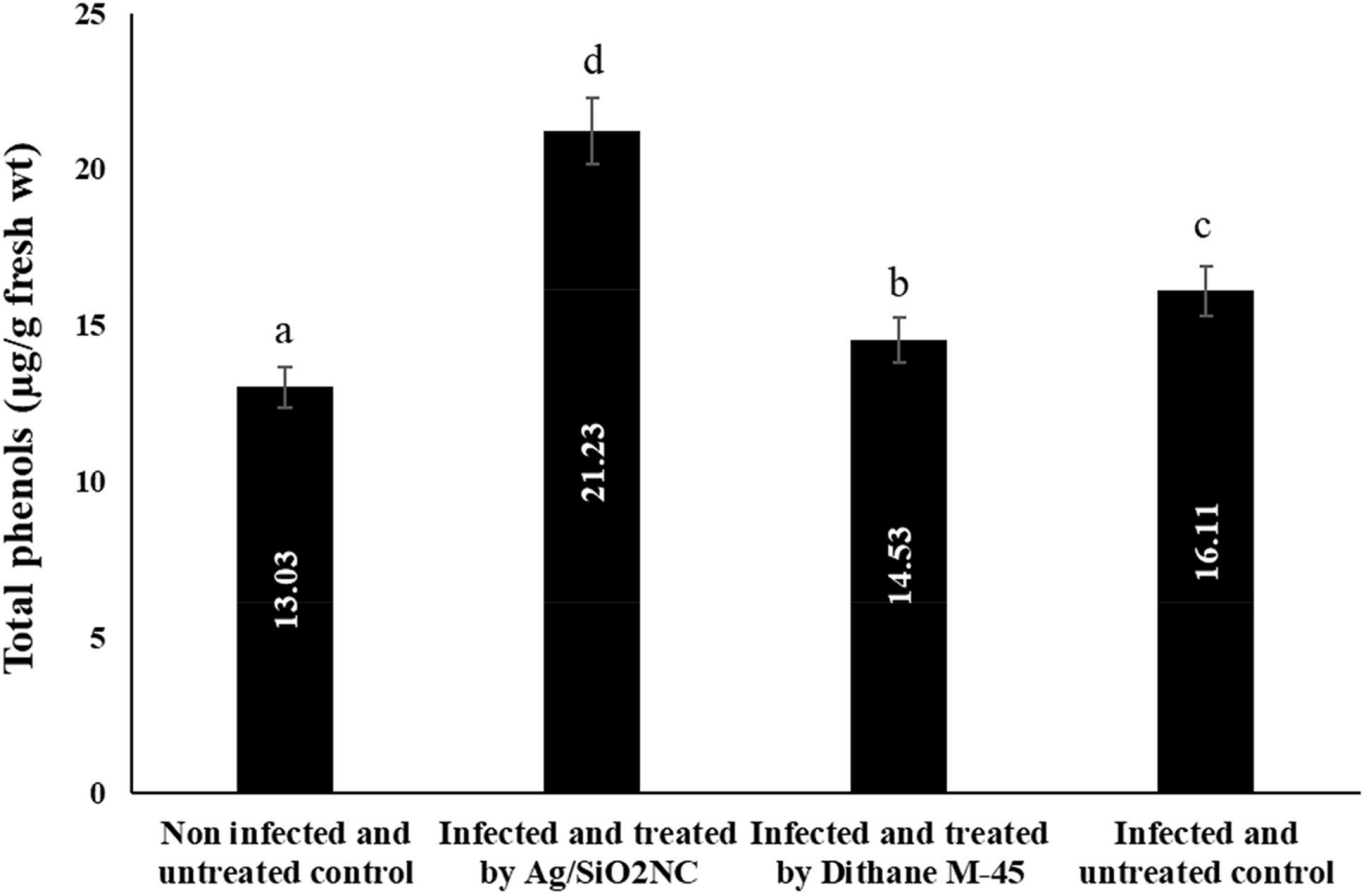
The total phenols in yielded seeds of faba bean plants (non-infected/infected) in the presence or absence of Ag/SiO_2_NC or Dithane M-45. Vertical bars represent the SE. Means denoted by similar letter are not significantly different at *p* ≤ 0.05 using Tukey–Kramer HSD test

While untreated infected plants showed a slight rise in phenol amount, Ag/SiO_2_NC-treated plants showed a great increase and stimulation of phenolic compounds (Fig. 5). Pathogen resulted in a massive decrease in the yielded faba bean seeds.

### Effect of Ag/SiO_2_NC on the activity of POD and PPO

**Fig. 6:**
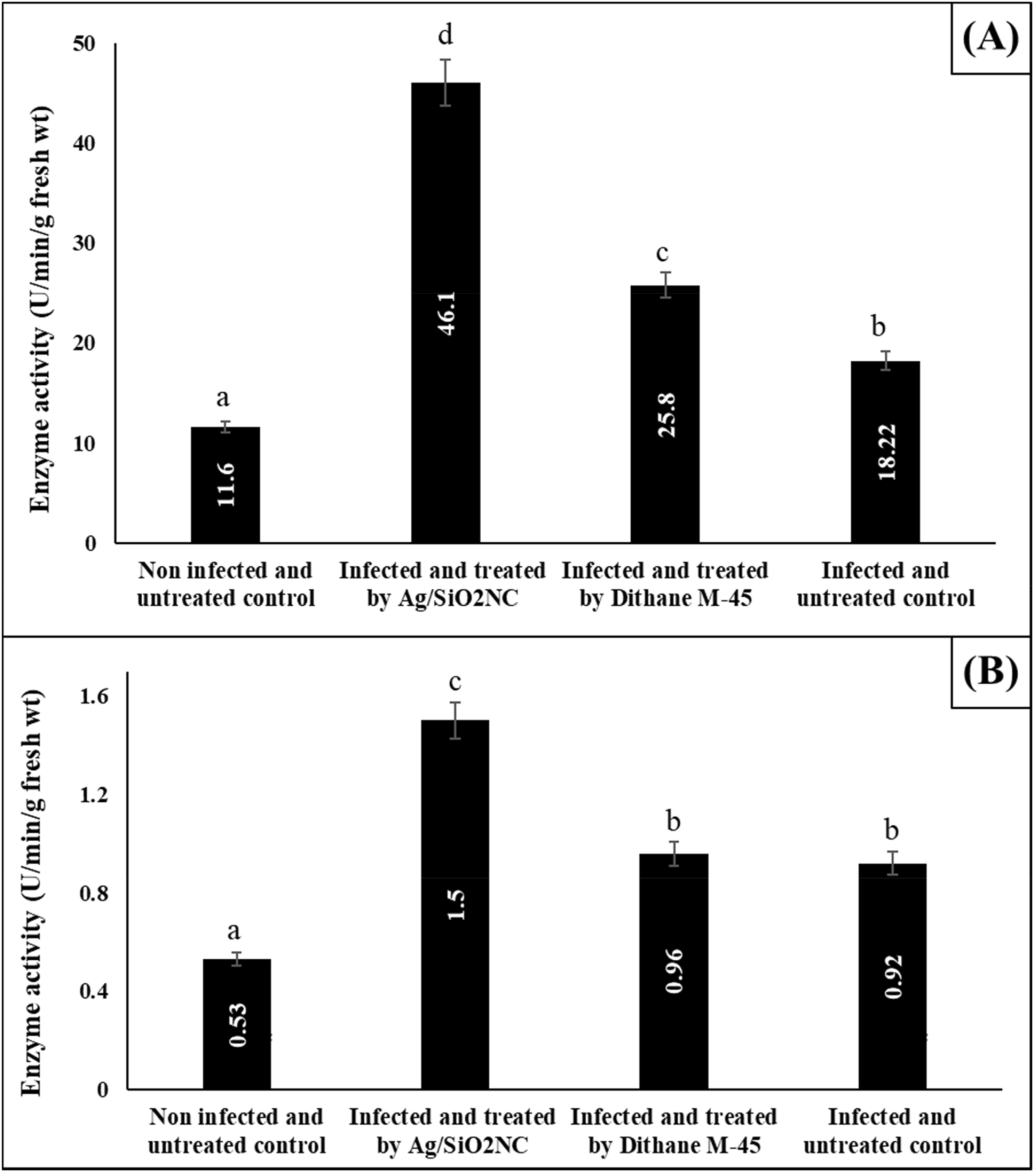
The activity of **A** POD and **B** PPO of faba bean plants (non-infected/infected) in the presence or absence of Ag/SiO_2_NC or Dithane M-45. Vertical bars represent the SE. Means denoted by similar letter are not significantly different at *p* ≤ 0.05 using Tukey–Kramer HSD test

The activities of the tested defence enzymes (POD and PPO) were significantly increased (*P* ≤ 0.05) by the development of the infection of faba bean plants when compared to the control one (Fig. 6). Besides, the treatment of faba bean plants with Ag/SiO_2_NC increased these enzyme activities much higher than the Dithane M-45-treated plants.

### Estimation of total protein of faba bean seeds

**Fig. 7:**
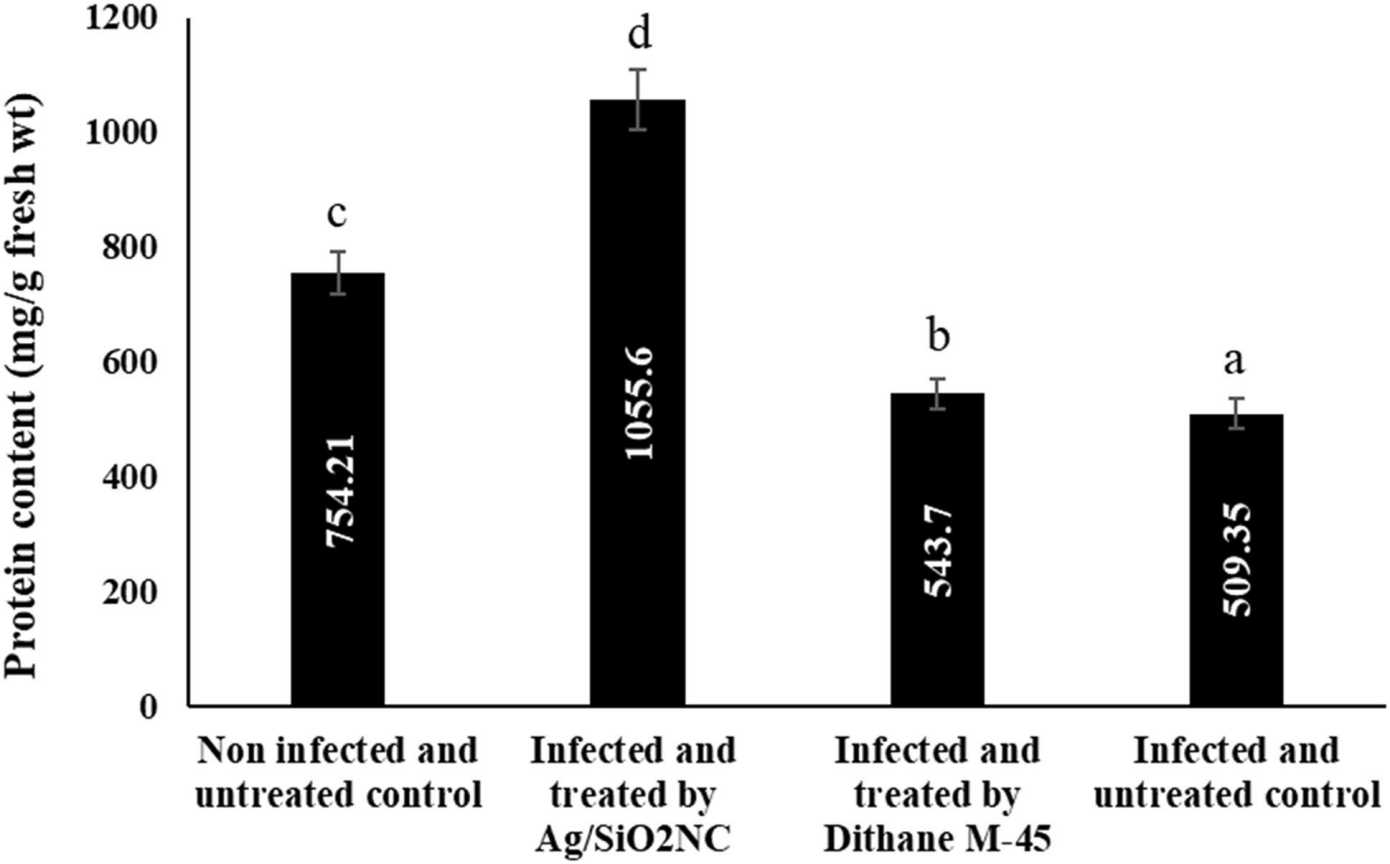
The total protein content in yielded seeds of faba bean plants (non-infected/infected) in the presence or absence of Ag/SiO_2_NC or Dithane M-45. Vertical bars represent the SE. Means denoted by similar letter are not significantly different at *p* ≤ 0.05 using Tukey–Kramer HSD test

The pathogen caused a significant reduction in the total protein content of the faba bean seeds. Furthermore, the total protein content of Ag/SiO_2_NC-treated seed increased significantly (*P* ≤ 0.05). Seed treated with Ag/SiO_2_NC completely prevented the negative effects of *B. cinerea* infection as compared to untreated infected plants. As shown in Fig. 7, Ag/SiO_2_NC-treated plants were more resistant to *B. cinerea* infection by inducing the accumulation of total phenols and total protein content in produced seeds as compared to Dithane M-45 treatment.

### Estimation of silver concentration content

**Fig. 8:**
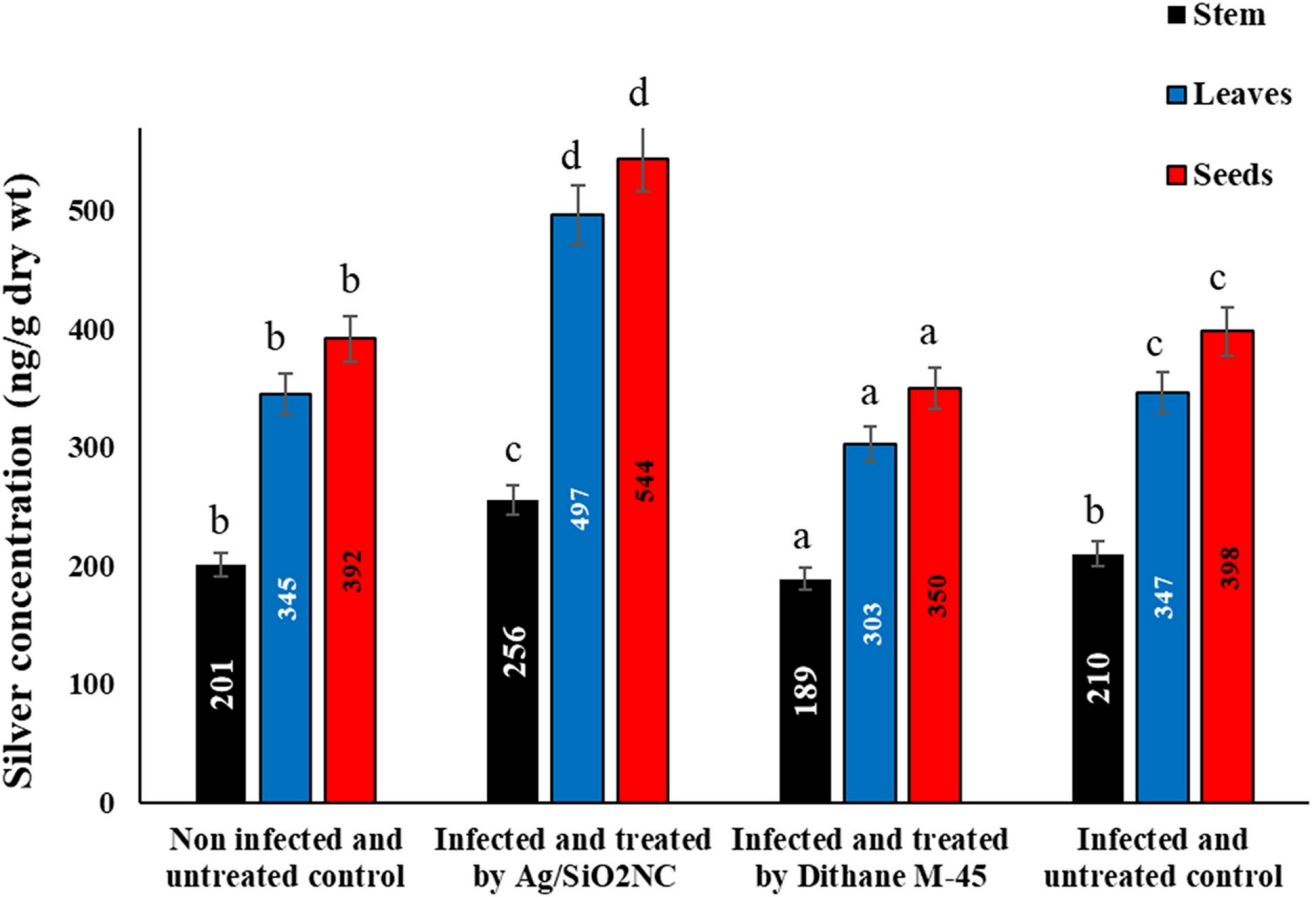
The silver content in faba bean stems, leaves and seeds of faba bean plants (non-infected/infected) in the presence or absence of Ag/SiO_2_NC or Dithane M-45. Vertical bars represent the SE. Means denoted by similar letter are not significantly different at *p* ≤ 0.05 using Tukey–Kramer HSD test

Ag/SiO_2_NC-treated plants displayed a slight incensement in the silver concentration of stems, leaves and seeds compared to the other plants (Fig. 8).

### Ultrastructural study

**Fig. 9:**
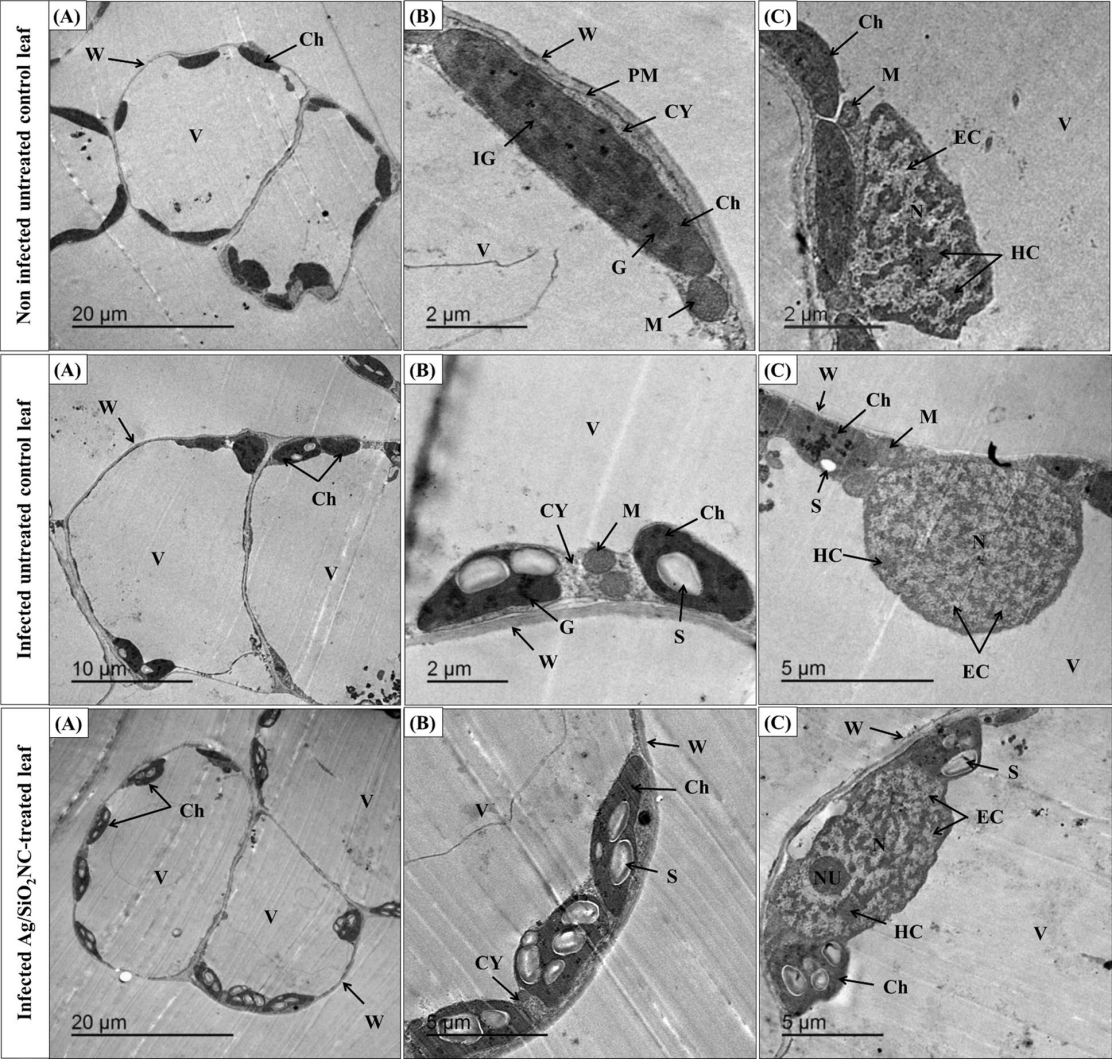
TEM micrographs of faba bean leaves (non-infected/infected) in the presence or absence of Ag/SiO_2_NC where **A** is a whole cells view with scale bar = 20 μm and **B**, **C** are a highly magnified part of chloroplasts and chloroplasts next to nucleus, respectively, with scale bar = 5 or 2 μm. *Ch* chloroplast, *S* starch grains, *N* nucleus, *HC* electron-dense heterochromatin, *EC* electron-lucent euchromatin, *W* cell wall, *CY* cytoplasm, *G* grana system, *IG* intergranal lamellae, *M* mitochondria, *NU* nucleolus and *V* vacuole

The data in Fig. 9 revealed that the leaves of non-infected untreated faba bean had a normal cell plasma membrane, ellipsoidal-shaped chloroplasts with an organized membrane system of grana and intergranal lamellae, mitochondria, big vacuole, and a thin cytoplasm. In addition, the chloroplasts were arranged close to the cell wall. On the other hand, the infected untreated faba bean leaves showed few numbers of round-shaped chloroplasts with an irregular membrane system. However, the Ag/SiO_2_NC-treated plants had normal thick cell walls, normal plasma membrane, big vacuole and well-organized chloroplasts with big starch grains. Furthermore, the nucleus of Ag/SiO_2_NC-treated plants had distinguished electron-dense heterochromatin, electron-lucent euchromatin and an obvious large nucleolus.

## Discussion

Various approaches have proved their ability to manage chocolate spot disease and reduce yield losses of faba bean all around the world. Several fungicides and chemical compounds are effective in combating this disease (Anil et al. 2013). The use of fungicides is unfavourable because of high costs, negative effects on human health and the environment, and the fact that it kills beneficial soil microflora (Arora et al. 2018). In this regard, the need to change sustain techniques and provide new ways is fundamental. Nanotechnology, particularly green innovation offers an impressive commitment to easing these challenges. It has prompted changes and advances in numerous technologies and can help to develop different fields of the agricultural sector such as fertilizers, fungicides, composts, and different industrial applications related to agriculture. Because of their novel properties, nanomaterials are considered potent antimicrobial agents and/or stabilizing transporters for fertilizers and pesticides, as well as working with controlled supplement transfer and aid in crop protection (Ashraf et al. 2021). Thus, this study aimed to supplant a safe, inexpensive, and effective cutting-edge biosynthesized fungicidal nanocomposite (Ag/SiO_2_NC) for controlling the chocolate spot disease of a faba bean plant, as well as to work on the growth of faba bean plants’ yield. The previous studies (Shah et al. 2014; Abd-Alla et al. 2016; Mahakham et al. 2017) tested and documented the enhancement and/or antimicrobial action of solo Si, Ag, nanosilicon or AgNPs on faba bean plants. While the presented study combined between AgNPs and Si to get the dual nutritional and antimicrobial action of both materials. The presented work is, to our knowledge, a first study for the in vivo antifungal activity of Ag/SiO_2_NC against of chocolate spot disease of *V. faba* L. caused by *B. fabae*.

The biosynthesized Ag/SiO_2_NC was characterized and showed a positive charge (+ 31.0 mV), as well as embedding of well-dispersed spherical-shaped AgNPs (12–29 nm). Rodrigues et al. (2020) demonstrated the biosynthesis of Ag/SiO_2_NC with AgNPs with a mean size of 45 ± 12 nm and a negative charge (− 35.5) using green tea extract. The positive charge of the nanomaterials increases the effective electrostatic association with the microbial cell wall's negative charges, allowing them to easily penetrate the cell membrane (El-Zahed et al. 2022). FTIR and XRD results stated the purity of Ag/SiO_2_NC particles that formed exclusively from Si, oxygen and silver, which was in concurrence with Jeon et al. (2003) and Wei et al. (2014) results.

Sand and clay soil provide the best vegetative growth for plants in terms of plant height, leaf number/plant, leaf dry weight, and high inflorescences (Nabih 1991; Mazhar et al. 2010; Abd El Gayed and Attia 2018). In the in vivo experiments, clay and sand were mixed in a ratio 2:1 volume-to-volume. Clay represents two extreme situations for agricultural reclamation compared to sand because of its strong water-holding, high productivity and cation exchange capabilities due to its smectite mineralogy (Million et al. 1987). These sand–clay combinations are advantageous for growing crops. Sandy soil helping the root system of the plant to benefit from all the nutrients provided to it as well as facilitates the penetration and spread of the root system that increase the aeration of the soil including oxygen levels. In addition, the absence of these elements will lead to the yellowing of plant leaves, the growth of improper fruits, or even the death of plants (Million et al. 1987; Ghareeb et al. 2021). In general, this study aimed to provide a practical and field model that simulates what the farmer faces during real cultivation to achieve maximum benefits and study the extent to which this study can be applied. The results showed that Ag/SiO_2_NC treatments significantly increased plant growth and yield compared to control. Plants growing under natural conditions do not suffer from Si deficiencies (such as the used control in field experiment). Although Si is considered as a non-essential element in plant nutrition (Richmond and Sussman 2003), several studies documented that the exogenous application of Si and its compounds can stimulate growth of most plant species and increase their yields (Romero-Aranda et al. 2006; Xie et al. 2014; Ismail et al. 2022). This effect of Si on plant growth is dose and crop specific. Generally, Si and its compounds such Ag/SiO_2_NC affects plant growth by affecting several parameters, including improvement the translocation of minerals and metabolites necessary for seed setting, upregulation of plant defense systems (Hasan et al. 2020), improvements in the ultrastructure of leaf organelles including an increasing in the chlorophyll contents, enlarging chloroplasts size and increasing number of grana in leaves resulting in improving photosynthetic potential and efficiency (Zhu et al. 2004), an enhancement in plant water status (Abou-Baker et al. 2012), and alleviation of the unfavorable and toxic ions in soil (Tahir et al. 2006). Also, Si treatments were reported to increase potassium ions uptake and decrease sodium ions uptake resulting in low electrolytic leakage and lipid peroxidation compared to control plants which is considered to be the major mechanism responsible for better growth and yield of plants (Al-aghabary et al. 2004).

Under *B. cinerea* infection, shoot length, shoot, and root fresh weight, shoot and root dry weight, and leaf area were decreased when compared with control, while these growth parameters were increased in case of treatment of plants by Ag/SiO_2_NC compared to that of the control. Under *B. cinerea* infection, shoot length, shoot, and root fresh weight, shoot and root dry weight, and leaf area were decreased when compared with control, while these growth parameters were increased in case of treatment of plants by Ag/SiO_2_NC compared to that of the control. Also, the registered data of plant yield revealed that *B. cinerea* infection decreased plant crop yield per plant including number of seeds per pod, seed weight per pod, total seed yield, and straw yield compared to control and Ag/SiO_2_NC-treated plants. The results showed that using Ag/SiO_2_NC on infected faba bean plants reduced chocolate spot disease symptoms considerably. In vitro, Ag/SiO_2_NC were shown to be more efficient inhibitors of *B. cinerea* (lower MIC values) than AgNO_3_ and SiO_2_ (Jeon et al. 2003; Wei et al. 2014; Abdul-Karim and Hussein 2022). *B. cinerea* caused a severe reduction in the shoot and root growth of faba bean plants (Mahmoud et al., 2011) which might be due to faba bean plant consumption by fungal hydrolysis enzymes that kill the infected plants (Elnahal et al. 2022). However, Ag/SiO_2_NC-treated plants showed a notable increase in both shoot and root growth parameters besides their yields. El-Flaah et al. (2021) and Hamed et al. (2019) documented the enhancement of the metabolically, physiological, and yield of faba bean throughout the treatment by nanosilicon and nanosilver, respectively. In addition, the use of Ag/SiO_2_NC increased shoot biomass compared to root biomass (Garg and Singh 2018). Qados (2015) reported the increase of proline content in the case of using nanosilicon in faba bean plants infected with *B. cinerea*. Similarly, Ag/SiO_2_NC enhanced the accumulation of proline in faba bean plants (Sarkar et al. 2022). Ag/SiO_2_NC treatment induced a great increase in the soluble phenolic compounds content (Farouk et al. 2017) and the protective antioxidant enzymes POD and PPO (Polanco et al. 2014). Also, Fortunato et al. (2015) reported an increase in the activity of POD and PPO when *B. cinerea*-infected soybean plants were treated with silicon. *B. cinerea* infection decreased the total content of protein in faba bean seeds (Rubiales and Khazaei 2022) in contrast to Ag/SiO_2_NC-treated plants that showed a significant increase in the protein content (Roohizadeh et al., 2014).

TEM micrographs showed several ultrastructural changes in host cell organelles after infection by *B. cinerea*. This pathogen produces hydrolytic enzymes that can degrade the cell wall (Elnahal et al. 2022), plasma membrane and middle lamella of plant cells (Kohmoto et al. 1993). Also, the mesophyll cells of infected plants had low numbers in chloroplasts (Farouk et al., 2017). On the other hand, Ag/SiO_2_NC treatment increased chloroplast number without abnormal effects and with large starch granules. In accordance, Asgari et al. (2018) used nanosilicon in oat plants and reported the normal ultrastructure of chloroplasts with normal grana.

High concentrations of Ag ions caused noticeable changes in treated plants, including the degradation of cytoplasmic components inside cells through autophagy and negative effects on the chloroplast ultrastructure. The fact that none of these changes were noticed after treating plants with Ag/SiO_2_NC points to the low rate at which AgNPs accumulated inside the treated plants. Additionally, compared to earlier researches (Abou-Baker et al. 2012; Shah et al. 2014; Abd-Alla et al. 2016; Mahakham et al. 2017), the current study found that the accumulation of Ag ions in the stem, leaves, and seeds was only 256, 497, and 540 ng/g dry wt, respectively. The present study demonstrated that the examined faba bean stem, leaf and yielded seeds using atomic spectrometer showed a slight increase in the concentration of silver content in Ag/SiO_2_NC-treated plants. This finding stated the low realizability of AgNPs from Ag/SiO_2_NC resulting in decreasing its accumulation in treated plants. In addition, SiO_2_ plays an important role in the reduction of the accumulation of harmful ion inside the plants (Hussain et al. 2019). Consequently, using SiO_2_ may be a method for decreasing the toxicity of AgNPs in plants and its concentration in grains. Comparing treated plants to untreated plants under abiotic stress, it can increase chlorophyll content, cause potassium ions uptake, modify sodium ions levels, and lessen cell wall damage (Hussain et al. 2019). SiO_2_ may also help in increasing of plants growth rate, biomass and productivity while experiencing less oxidative stress. These results are promising outcomes for the application of the biosynthesized silver nanocomposite as a safe and effective antifungal agent against *B. cinerea*, as well as limiting the negative and adverse harmful effects caused by the accumulation of silver ions in plants, which was limiting the use of these nanoparticles for fear of silver intoxication. Nevertheless, the toxicity of the biosynthesized silver nanocomposite needs for further research to explore its toxicity using in vivo with an animal model in future work.

## Conclusions

The current study revealed that silver/silicon dioxide nanocomposite (Ag/SiO_2_NC) may be used as nutrients, antifungals, and growth and yield promoters in a variety of plants, including faba bean. Furthermore, the results of this study validated the effect of Ag/SiO_2_NC in suppressing chocolate spot disease of the faba bean caused by *B. cinerea* by improving physiological and ultrastructural features. In addition, Ag/SiO_2_NC improved faba bean resistance to *B. cinerea* by increasing proline, phenols, and defense enzymes (peroxidase and polyphenol oxidase enzymes). Furthermore, the toxicity of Ag/SiO_2_NC needs to be verified in vivo with an animal model.

## Data Availability

The datasets used and/or analysed during the current study are available from the corresponding author on reasonable request.

## Abbreviations

Si: Silicon
SiO_2_: Silicon dioxide
AgNPs: Silver nanoparticles
Ag/SiO_2_NC: Silver/silicon dioxide nanocomposite
UV–Vis: Ultraviolet–visible
FTIR: Fourier transform-infrared spectroscopy
XRD: The X-ray diffraction pattern
TEM: Transmission electron microscopy
MIC: The minimal inhibition concentration
FDA: The faba bean dextrose agar
FDB: The faba bean dextrose broth
POD: Peroxidase activity
PPO: Polyphenol oxidase activity
ANOVA: One-way analysis of variance
